# Supplementary material for: Effect of Graphene Sheets Embedded Carbon Films on the Fretting Wear Behaviors of Orthodontic Archwire–Bracket Contacts
Source: Nanomaterials (Basel). 2022 Sep 30;12(19):3430. doi: 10.3390/nano12193430 (PMC9565541; doi:10.3390/nano12193430)
Supplement: Supplementary file 1 [file nanomaterials-12-03430-s001.zip › nanomaterials-1902523-supplementary.pdf]

nanomaterials

Article

Effect of Graphene Sheets Embedded Carbon Films on the Fretting Wear Behaviors of Orthodontic Archwire–Bracket Contacts

Pengfei Wang *, Xin Luo, Jiajie Qin, Zonglin Pan and Kai Zhou

Institute of Nanosurface Science and Engineering (INSE), Guangdong Provincial Key Laboratory of Micro/Nano Optomechatronics Engineering, College of Mechatronics and Control Engineering, Shenzhen University, Shenzhen 518060, China

* Correspondence: wangpf@szu.edu.cn; Tel.: +86-755-2267-3825

Citation: Wang, P.; Luo, X.; Qin, J.; Pan, Z.; Zhou, K. Effect of Graphene Sheets Embedded Carbon Films on the Fretting Wear Behaviors of Orthodontic Archwire–Bracket Contacts. *Nanomaterials* **2022**, *12*, 3430. https://doi.org/10.3390/nano12193430

Academic Editors: Jin-Hae Chang and Marcelo Antunes

Received: 22 August 2022
Accepted: 27 September 2022
Published: 30 September 2022

Publisher's Note: MDPI stays neutral with regard to jurisdictional claims in published maps and institutional affiliations.

Copyright: © 2022 by the authors. Licensee MDPI, Basel, Switzerland. This article is an open access article distributed under the terms and conditions of the Creative Commons Attribution (CC BY) license (https://creativecommons.org/licenses/by/4.0/).

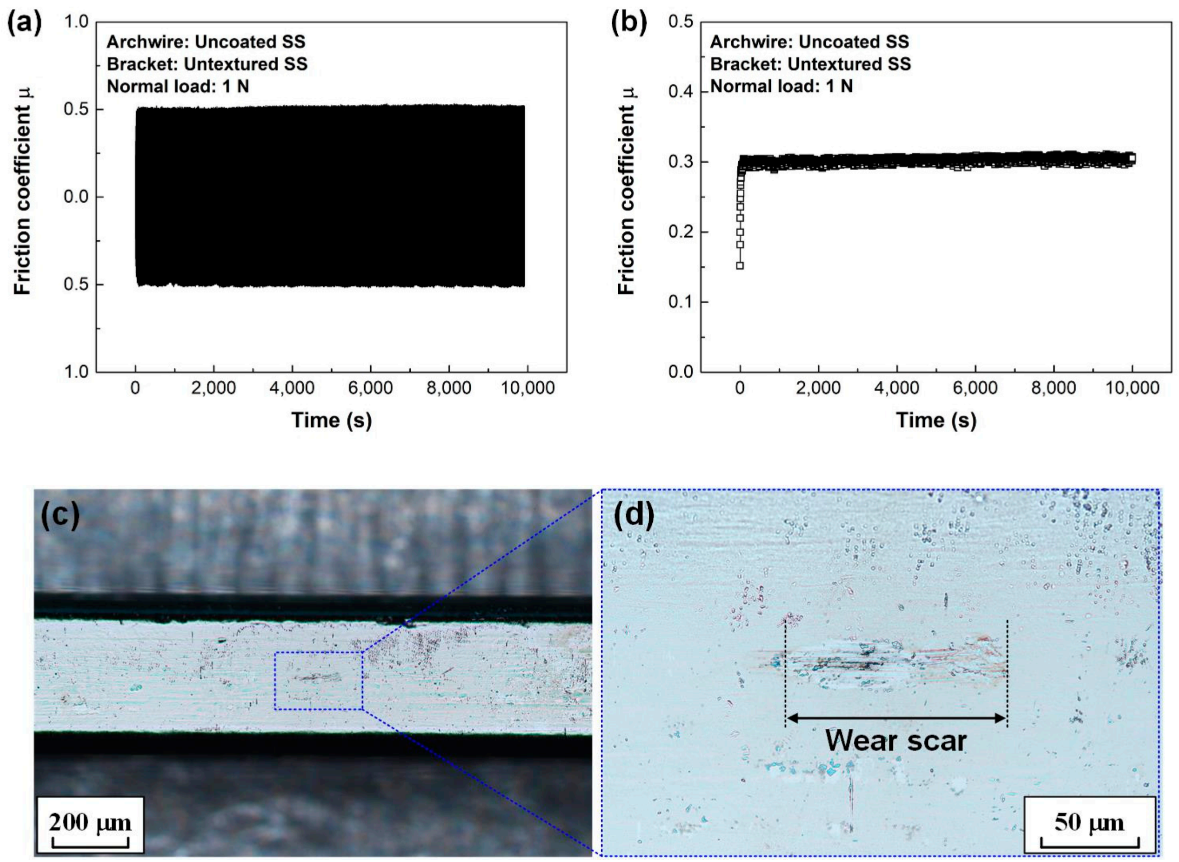

Figure S1. Fretting wear test of uncoated stainless steel archwire sliding against conventional untextured stainless steel bracket under artificial saliva environment. (**a**) Friction curve. (**b**) Average friction coefficient. (**c**) Optical microscopy image of the wear scar on the archwire. (**d**) Enlargement optical microscopy image of the selected rectangular area in (c).

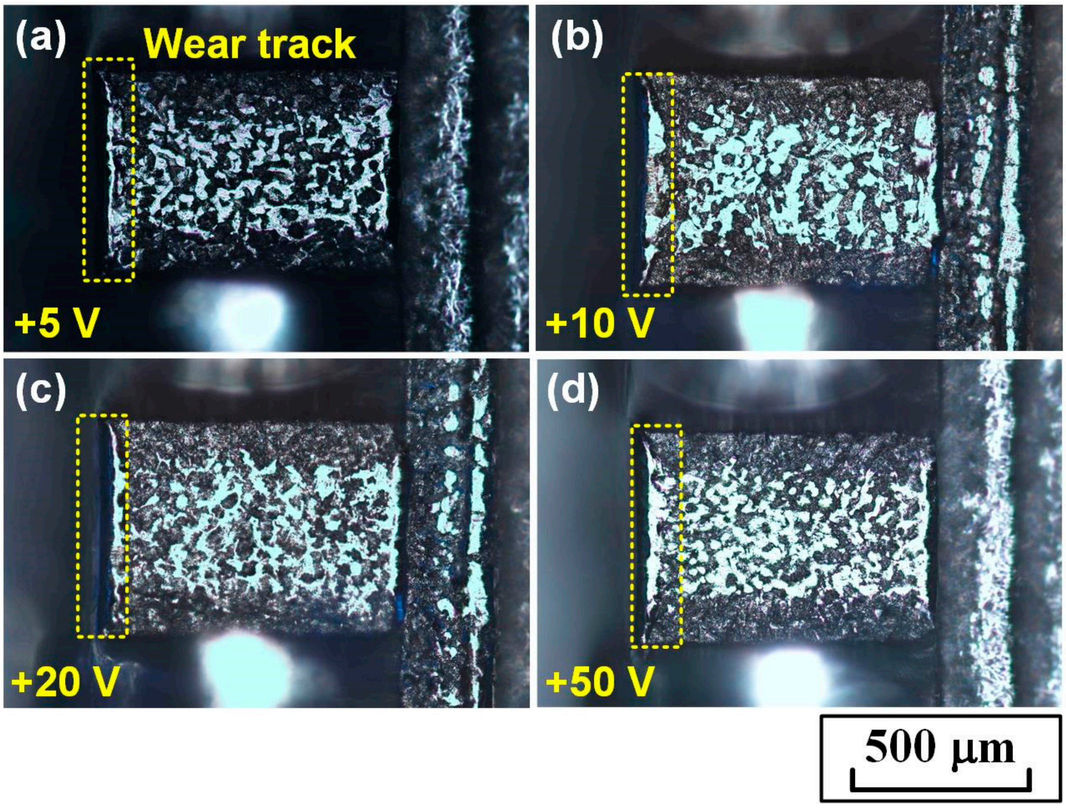

Figure S2. Optical microscopy images of wear tracks on the slot surfaces of untextured stainless steel brackets after running against different carbon films coated stainless steel archwires under artificial saliva environment for 10,000 seconds. (**a**) +5 V, (**b**) +10 V, (**c**) +20 V, and (**d**) +50 V. The wear tracks generally located at the edge of the slot surfaces are denoted by yellow dotted rectangular.

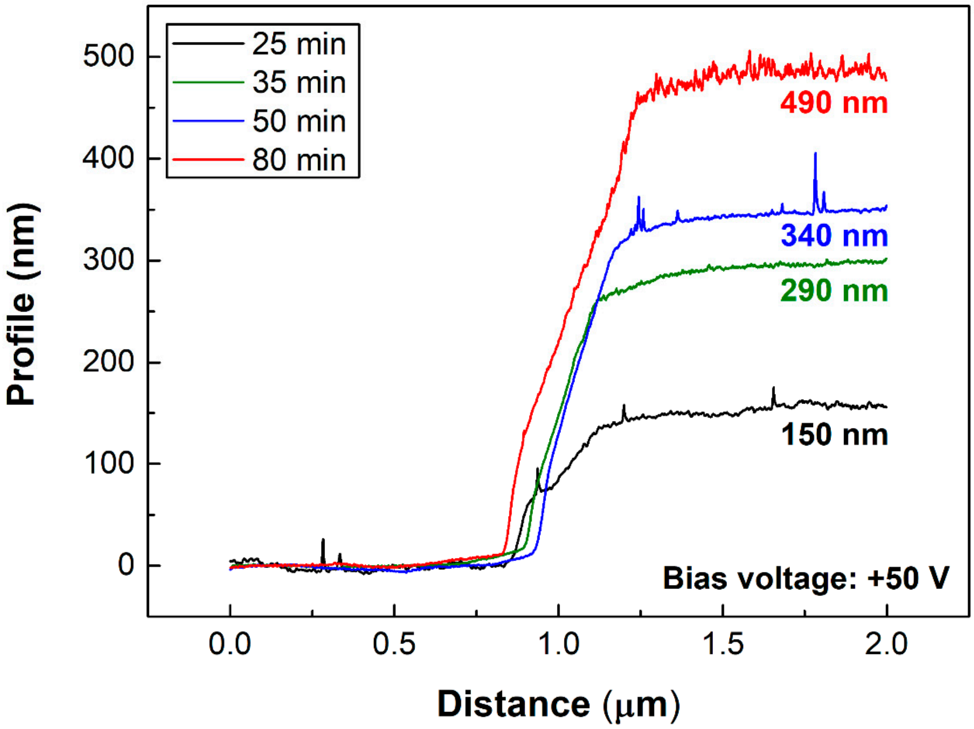

Figure S3. Cross-sectional profiles of carbon films fabricated under substrate bias voltage of +50 V with different deposition time for calculating the film thickness. Carbon films were deposited simultaneously on both the stainless steel archwires and Si substrate under the same deposition parameters. The thickness of the carbon films deposited on the Si substrate was measured by using a surface profiler (Dektak-XT, Bruker, San Jose, CA, USA). The deposition rate calculated from the carbon film thickness and deposition time varied from 6.0 to 8.3 nm/min.

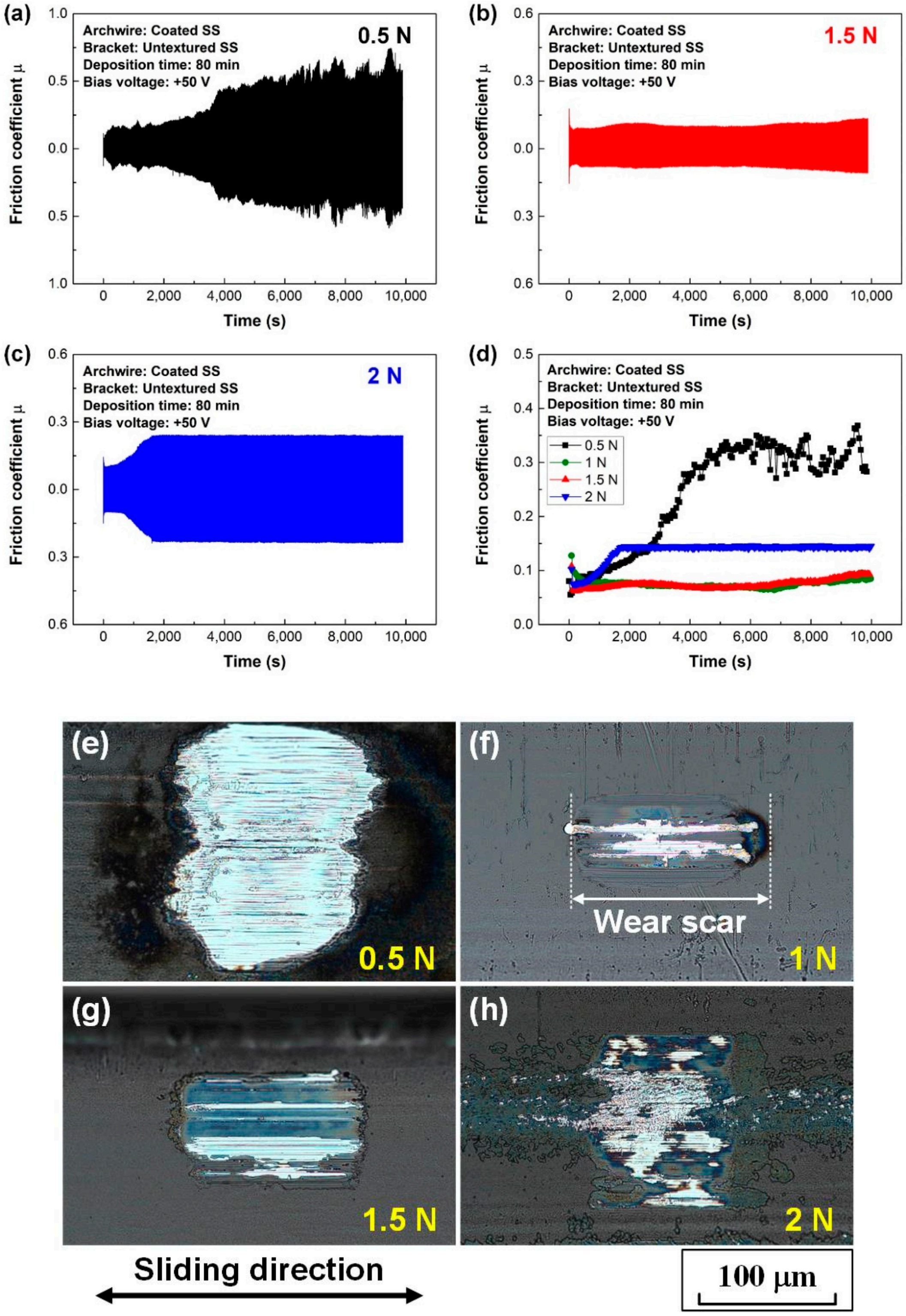

Figure S4. Fretting wear results of carbon film (+50 V and 80 min) coated archwires sliding against brackets under different normal loads in artificial saliva environment. Friction curves with normal load of (**a**) 0.5 N, (**b**) 1.5 N, and (**c**) 2 N. (**d**) Average friction coefficient under different normal loads. Optical microscopy images of the wear scars on the archwires with normal load of (**e**) 0.5 N, (**f**) 1 N, (**g**) 1.5 N, and (**h**) 2 N.

.

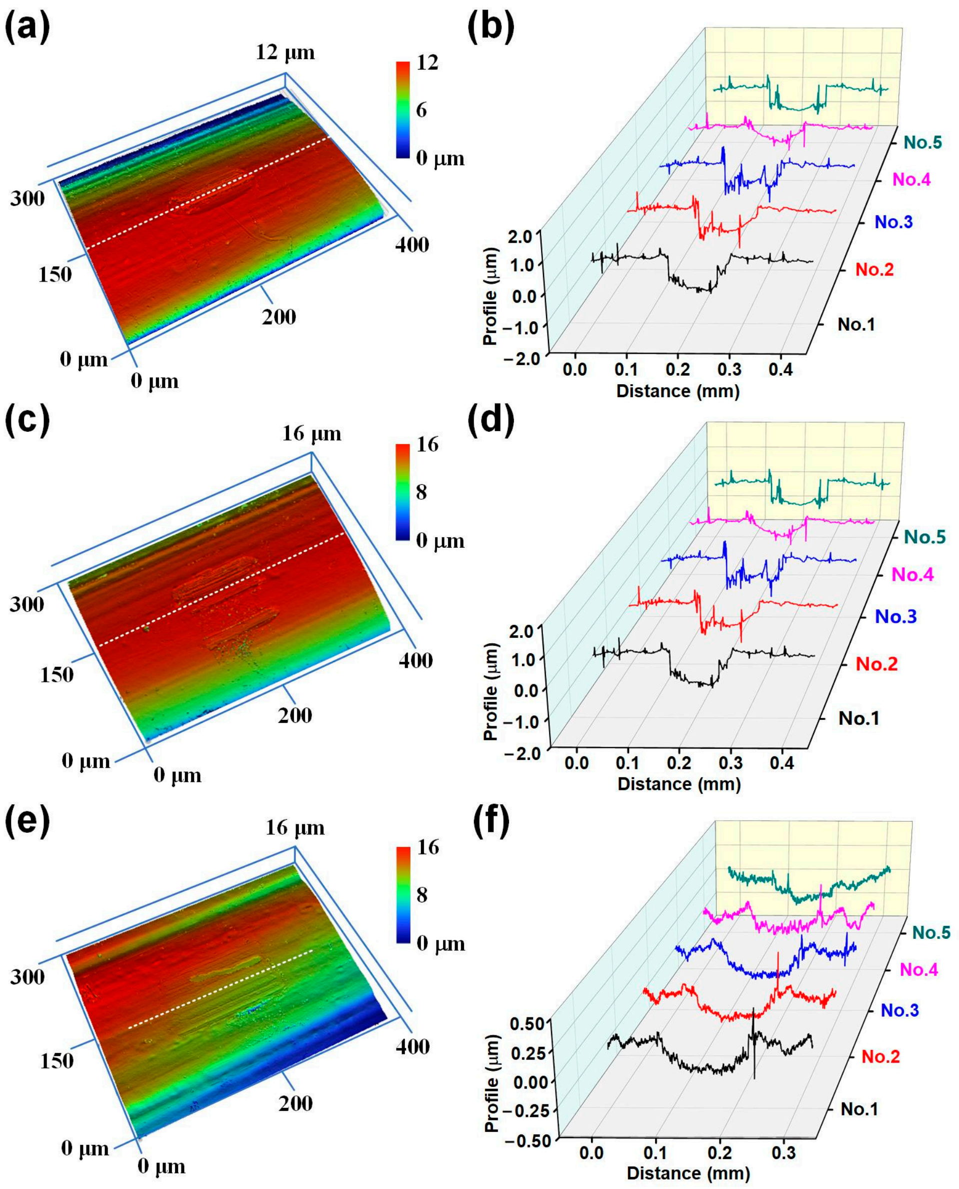

Figure S5. Surface characterization of the wear scars on the GSEC film coated archwires after sliding against untextured and micro-groove textured stainless steel brackets for 10,000 times in artificial saliva environment. (**a**) 3D image and (**b**) 2D cross-sectional profile of the wear scars on the GSEC film (+50 V and 80 min) coated archwires after running against untextured bracket (denoted as L0). (**c**) 3D image and (**d**) 2D cross-sectional profile of the wear scars on the GSEC film (+50 V and 80 min) coated archwire after running against one-row micro-groove textured bracket (denoted as L1). (**e**) 3D image and (**f**) 2D cross-sectional profile of the wear scars on the GSEC film (+50 V and 80 min) coated archwire after running against two-row micro-groove textured bracket (denoted as L2).
